# Factors associated with the onset of Alzheimer's disease: Data mining in the French nationwide discharge summary database between 2008 and 2014

**DOI:** 10.1371/journal.pone.0220174

**Published:** 2019-07-25

**Authors:** Michaël Rochoy, Régis Bordet, Sophie Gautier, Emmanuel Chazard

**Affiliations:** 1 Univ. Lille, Lille, France; 2 INSERM, U1171-Degenerative and Vascular Cognitive Disorders, Lille, France; 3 EA2694, Public Health Department, Lille, France; University of Toronto, CANADA

## Abstract

**Introduction:**

Identifying modifiable risk factors for Alzheimer’s disease (AD) is critical for research. Data mining may be a useful tool for finding new AD associated factors.

**Methods:**

We included all patients over 49 years of age, hospitalized in France in 2008 (without dementia) and in 2014. Dependent variable was AD or AD dementia diagnosis in 2014. We recoded the diagnoses of hospital stays (in ICD-10) into 137 explanatory variables.To avoid overweighting the "age" variable, we divided the population into 7 sub-populations of 5 years.

**Results:**

We analyzed 1,390,307 patients in the PMSI in 2008 and 2014: 55,997 patients had coding for AD or AD dementia in 2014 (4.04%). We associated Alzheimer disease in 2014 with about 20 variables including male sex, stroke, diabetes mellitus, mental retardation, bipolar disorder, intoxication, Parkinson disease, depression, anxiety disorders, alcohol, undernutrition, fall and 3 less explored variables: intracranial hypertension (odd radio [95% confidence interval]: 1.16 [1.12–1.20] in 70–80 years group), psychotic disorder (OR: 1.09 [1.07–1.11] in 70–75 years group) and epilepsy (OR: 1.06 [1.05–1.07] after 70 years).

**Discussion:**

We analyzed 137 variables in the PMSI identified some well-known risk factors for AD, and highlighted a possible association with intracranial hypertension, which merits further investigation. Better knowledge of associations could lead to better targeting (identifying) at-risk patients, and better prevention of AD, in order to reduce its impact.

## Introduction

In 2015, the global prevalence of dementia was re-estimated to 46 million people based on data from the Global Burden of Disease Study [1]. This number would exceed 115 millions in 2050 [2]. Alzheimer's dementia (AD) accounts for 60 to 70% of dementias [3,4]. There is a long presymptomatic period of about 15 years between biochemical changes in the brain and the development of AD [5,6]. About one-third of AD cases can be attributed to a modifiable cause [7]. Research of modifiable risk factors is a critical issue in dementia research: recent reviews of systematic reviews and meta-analyses have examined about 80 risk factors for AD [8,9]. Data mining is another tool for finding new associated factors.

Our aim was to determine factors associated with the occurrence of AD by using data mining in the database of all hospital stays in France (PMSI).

## Materials and methods

### Study design

We utilized the PMSI database (presented below). All the inpatient stays in 2008 and 2014 were included.

### Ethics statement

Approval from the French data protection agency (CNIL) was obtained to conduct the present study; the data were captured through the Technical Agency for Information on Hospital Care (ATIH), according to the current legislation. Studies assessing the accuracy of diagnosis coding by medical chart review are authorized by Lille University Hospital ethical committee.

### Data source

The PMSI database *(Programme de Médicalisation des Systèmes d’Information)* is the French nationwide exhaustive hospital discharge database [10]. Database used in our study comprehends all the inpatient stays, from nonprofit and for-profit acute care hospitals (medicine, surgery and obstetrics), excluding psychiatric hospitals and rehabilitation care centers. This database includes administrative data (admission and discharge dates and modes), demographic data (age, gender, geographic area), diagnoses encoded in ICD-10 [11], medical procedure encoded with the French medical classification for clinical procedures (CCAM: *Classification Commune des Actes Médicaux*) [12], and other pieces of information [13]. This information is anonymized and can be reused for research purposes [14].

The database comprehends 23,781,314 inpatient stays in 2008 and 27,087,492 in 2014.

### Inclusion and exclusion criteria

We included all patients present in both the 2008 and 2014 PMSI databases who were over 49 years of age. We excluded patients with dementia in 2008.

Dementia and related diseases encoding rules have been defined in 2006 [15]. In accordance with those rules, the inpatient stays having one of the following codes in 2008 were excluded (ICD-10 codes in brackets): AD (G30*, 4 codes), AD dementia (F00*, 84 codes), vascular dementia (F01*, 126 codes), other dementia (F02*, 120 codes), unspecified dementia (F03*, 20 codes), or mild cognitive impairment (F067*, 2 codes).

### Dependent and explicative variables

Dependent variable was AD in 2014, defined as AD (G30*) and AD dementia (F00*).

Sex and age (in 2008) were explicative variables available in the PMSI database. We created a "longitude" variable and a "latitude" variable from the prefectures of the departments where patients were hospitalized in 2008 (excluding the overseas departments and territories).

Diagnoses of inpatient stays in 2008 were recoded into binary variables after mapping the ICD-10 and the CCAM. Of the 40,109 ICD-10 codes, 11,768 were coded into 130 binary variables of interest (based on a literature review); of the 8,982 CCAM codes, 320 were coded into 10 binary variables. A total of 137 different variables were tested. The same code could correspond to several binary variables (for example "tuberculous meningitis" to "meningitis", "tuberculosis", "bacterial infection"). We aggregated data (ICD-10 and CCAM) from several hospital stays for the same patient.

### Statistical analysis

Before performing the data mining, we selected the variables. In order to avoid overweighting the "age" variable, we divided the population into 7 strata: 49–55 years (excluding day of 55 years), 55–60 years, 60–65 years, 65–70 years, 70–75 years, 75–80 years, 80 years and over.

In each strata, we ranked the 20 explanatory variables most associated with the onset of AD in 2014, using the "importance" value (*varImpPlot* function) of the random forest algorithm, based on the Breiman and Cutler Fortran code (package ‘*randomForest’*, version 4.6–12) [16]. Random Forest produced 20 classification trees (ntree) on a random fraction of the data, with 2 variables tested (*mtry*) at each division.

We then looked for interactions between these variables using decision trees by age group (package ‘rpart’ for Recursive Partitioning and Regression Trees, version 4.1–10) [17].

Finally, in each age group, we created a multivariate model by logistic regression, by age group, using a stepwise procedure (sequential replacement), with the 20 explanatory variables most associated with the onset of Alzheimer's disease in 2014 by importance (*randomForest*), as well as age, sex, longitude and latitude. The results of the logistic regression were expressed as odds ratio (OR) and 95% confidence interval. Statistics were computed using R version 3.3.2 [18].

## Results

### Characteristics of the population

**Fig 1:**
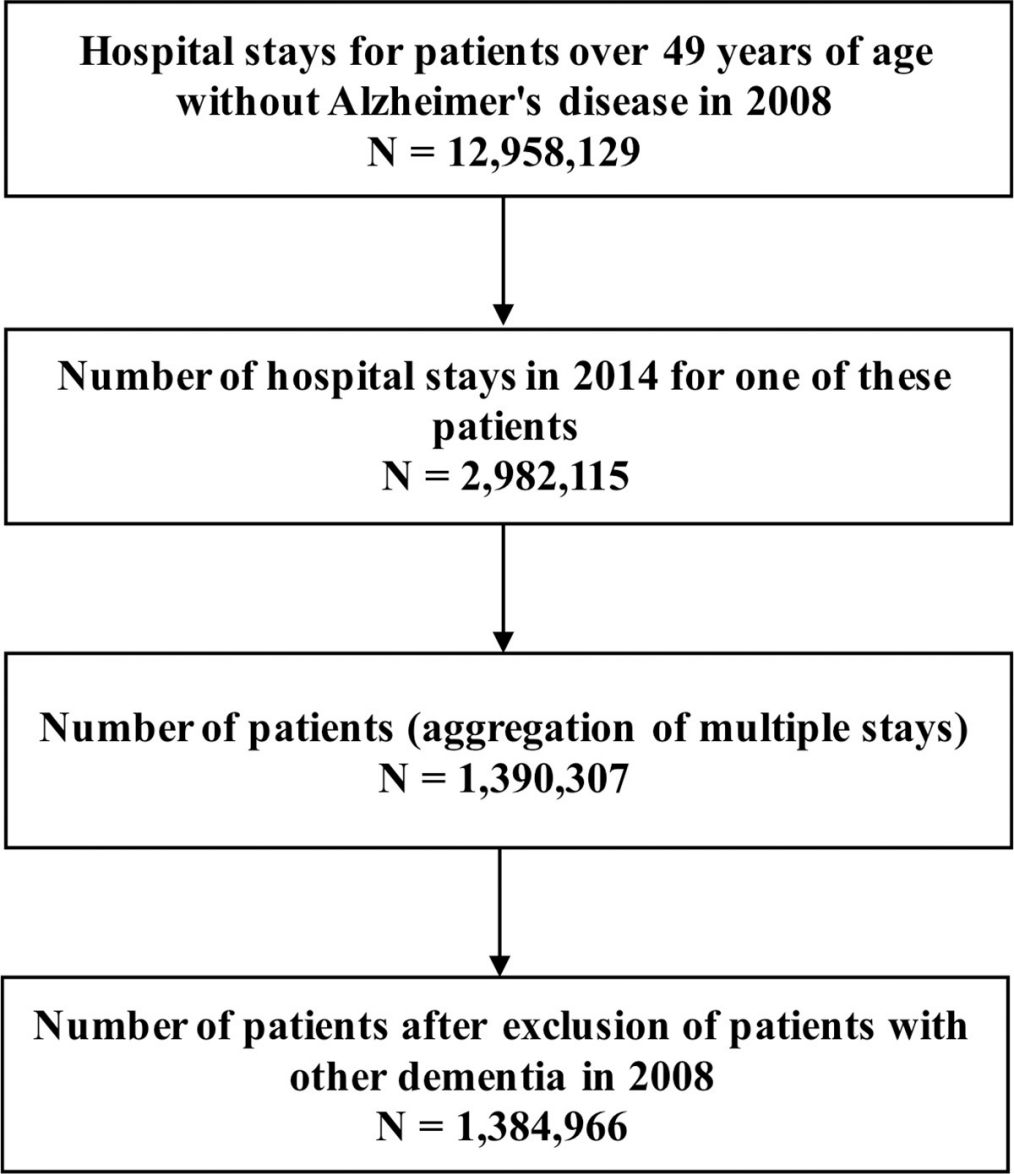
Flowchart.

We analyzed 1,390,307 patients in the PMSI in 2008 and 2014, without dementia in 2008 and aged 49 years or over on January 1, 2008 (**Fig 1**). The patients included were 66.7 ± 10.45 years of age on average. The main characteristics of patients in 2008 are described in **Table 1.**

**Table 1:** Characteristics of the 1,384,966 patients analyzed in 2008.

**Characteristics**	**Number**	**%**
Male sex	678 310	48.80
High blood pressure	387 037	27.84
Diabetes mellitus	178 803	12.86
Cancer	175 122	12.6
Visual impairment	151 828	10.92
Carcinomas	139 567	10.08
Non-ischemic heart disease	134 910	9.77
Acute coronary syndrome	131 167	9.43
Osteoarthritis	107 507	7.76
Diverticulosis	98 262	7.08
Obesity	96 276	6.93
Hernias (inguinal, crural, umbilical, abdominal)	80 283	5.79
Atrial fibrillation	75 432	5.43
Ulcer disease	71 035	5.12
Ischemic stroke	51 176	3.68
Disorders related to tobacco abuse	49 478	3.60
Anemia	49 718	3.58
Depression	46 706	3.31
Excess of alcohol	44 601	3.20
Family history of cancer	44 906	3.20
Heart failure	42 068	3.03
Sleep disorders	40 245	2.91
Hypothyroidism	36 572	2.63
Valvulopathy	35 105	2.54
Chronic venous insufficiency	26 225	1.89
Chronic hepatitis and cirrhosis	22 139	1.62
Martial deficiency	21 353	1.54
Inflammatory syndrome	21 105	1.52
Hydroelectrolytic disorder	19 343	1.43
Hearing impairment	16 999	1.22
Epilepsy	14 367	1.03
Rheumatoid arthritis	13 365	0.97
Intoxication	12 608	0.91
Fall	10 031	0.74
Undernutrition	10 092	0.73
Parkinson's disease	8 611	0.66
Pulmonary embolism	9 005	0.65
Chronic inflammatory bowel disease	8 478	0.61
Hypotension	6 911	0.50
Lymphoma	6 891	0.50
Leukaemia	4 191	0.50
Non-extrapyramidal movement disorders	6 654	0.48
Hyperthyroidism	5 445	0.39
Haemorrhagic stroke	4 885	0.35
HIV infection	4 532	0.33
Gout	4 618	0.33
Dyscalcemia	4 362	0.31
Psychotic disorder	4 204	0.31
Bipolar disorder	3 471	0.26
Psoriasis	3 589	0.26
Migraine	3 148	0.23
Tuberculosis	3 193	0.23
Vitamin D deficiency	3 108	0.22
Myeloma	2 593	0.19
History of brain surgery	2 672	0.19
Pituitary pathology	2 489	0.18
Non-migraine headaches	1 991	0.14
Immune deficiency	2 013	0.14
Intracranial hypertension	1 817	0.13
Vitamin B12 deficiency	1 669	0.12
Complex regional pain syndrome	1 629	0.12
Staphylococcus infection	1 405	0.10
VZV infection	1 177	0.09
Encephalitis	1 030	0.079
Vitamin B9 deficiency	1 001	0.07
Mental retardation	963	0.07
CNS Tumour	798	0.058
Myasthenia	746	0.05
Splenectomy	607	0.04
CMV infection	390	0.028
Family history of mental retardation	136	0.0001
Chlamydia infection	134	0.0001
Family history of alcoholism	69	0.00005

In our population, 55,997 patients had coding for AD or AD dementia in 2014 (4.04%).

Among 204,202 patients aged 49–55 years in 2008, 346 had AD coding in 2014 (0.17%); the rate gradually increased for the following strata: 680/207.513 (0.33%) for the 55–60 strata, 2,359/212,930 (0.64%) for the 60–65 strata, 3,337/190,433 (1.75%) for the 65–70 strata, 8,085/203,605 (3.97%) fort the 70–75 strata, 15,434/186,413 (8.28%) for the 75–80 strata and 26,756/179,870 (14.88%) for the 80 years and over strata.

### Multivariate models

In our models, some variables were significantly associated with the occurrence of AD in 2014 (**Table 2**). Variables changed according to the patient age, and included psychotic disorder (in the 65–70 and 70–75 years groups), intracranial hypertension (in the 70–75 and 75–80 years group), epilepsy (in the 70–75, 75–80 and over 80 years groups). Some appeared more aged-related as hemorrhagic stroke for the 70–75 years group; mental retardation and undernutrition for the 75–80 years group; depression and fall in over 80 years group.

**Table 2:** Odds-ratios [95% confidence interval] in multivariate models: factors associated (in red) and factors inversely associated (in blue) with the onset of Alzheimer's disease in 2014 (stronger associations at +/- 5% are in bold).

OR / Age (in 2008)	49–55	55–60	60–65	65–70	70–75	75–80	80+
**Male sex**	1.0005 [1.00009–1.0009]		0.999 [0.998–1.00]	0.997 [0.996–0.998]	0.992 [0.991–0.993]	0.978 [0.976–0.981]	0.965 [0.961–0.969]
**Ischemic stroke**		1.004 [1.003–1.006]	1.003 [1.002–1.006]				
**Hemorrhagic stroke**					**1.05 [1.04–1.07]**		
**Diabetes mellitus**		1.002 [1.001–1.003]		1.01 [1.01–1.01]	1.01 [1.01–1.01]		
**Mental retardation**		1.03 [1.02–1.03]	1.03 [1.02–1.04]	1.04 [1.01–1.07]		**1.10 [1.01–1.19]**	
**Bipolar disorder**			1.01 [1.00–1.02]	1.02 [1.00–1.03]	1.04 [1.02–1.06]		
**Psychotic disorder**	1.01 [1.01–1.02]		1.02 [1.01–1.03]	**1.07 [1.05–1.08]**	**1.09 [1.07–1.11]**	**1.12 [1.09–1.15]**	**1.14 [1.09–1.19]**
**Intoxication**	1.002 [1.001–1.003]		1.01 [1.00–1.01]	1.02 [1.01–1.02]	1.03 [1.02–1.04]		
**Parkinson**				1.03 [1.02–1.03]	1.02 [1.02–1.03]	1.03 [1.02–1.05]	
**Depression**				1.02 [1.02–1.03]	1.04 [1.03–1.05]	1.04 [1.04–1.05]	**1.06 [1.05–1.07]**
**Anxiety disorders**				1.01 [1.00–1.01]	1.03 [1.02–1.03]	1.04 [1.03–1.06]	
**Intracranial hypertension**			1.01 [1.00–1.02]	**1.07 [1.05–1.09]**	**1.08 [1.05–1.11]**	**1.16 [1.12–1.20]**	
**Alcohol**		1.003 [1.002–1.004]		1.02 [1.02–1.03]	1.03 [1.02–1.04]	1.04 [1.02–1.05]	
**Epilepsy**	1.008 [1.006–1.009]	1.02 [1.02–1.02]	1.02 [1.02–1.03]	1.04 [1.03–1.04]	**1.06 [1.05–1.07]**	**1.05 [1.04–1.07]**	**1.06 [1.04–1.08]**
**Undernutrition**				1.02 [1.01–1.02]	1.03 [1.01–1.04]	**1.05 [1.04–1.07]**	**1.05 [1.03–1.06]**
**Fall**	1.004 [1.002–1.008]					1.04 [1.03–1.05]	**1.05 [1.04–1.06]**
**Hypotension**						1.03 [1.02–1.05]	1.03 [1.01–1.05]
**Diverticulosis**	0.9986 [0.9977–0.9995]	0.9987 [0.9977–0.9997]	0.998 [0.997–0.9996]	0.996 [0.993–0.998]			
**Cancer / Malign tumor**				0.997 [0.995–0.999]		0.99 [0.98–0.99]	0.98 [0.97–0.99]
**Carcinoma / Benign tumor**					0.99 [0.99–0.99]		0.98 [0.97–0.99]
**Family history of cancer**		0.998 [0.997–0.999]		0.994 [0.991–0.998]	0.99 [0.98–1.00]	0.97 [0.96–0.98]	
**Other Movement Disorders**					0.99 [0.97–1.00]	0.96 [0.94–0.98]	**0.94 [0.91–0.97]**
**Immune Deficiency**			0.99 [0.98–1.00]				**0.90 [0.85–0.96]**
**Hernias**			0.997 [0.996–0.999]				0.98 [0.97–0.99]
**Rheumatoid Arthritis**				0.99 [0.99–1.00]	0.99 [0.98–1.00]		
**Lymphoma**						0.97 [0.95–0.99]	0.95 [0.92–0.98]
**Arthrosis**						0.99 [0.98–0.99]	0.98 [0.98–0.99]
**Heart failure**							0.99 [0.98–1.00]
**Inflammation**							0.97 [0.96–0.99]
**Acute coronary syndrome**							0.98 [0.98–0.99]
**Other Heart Diseases**							0.99 [0.99–1.00]
**Obesity**						0.98 [0.98–0.99]	0.97 [0.96–0.98]
**Valvulopathy**							0.99 [0.98–1.00]
**Atherosclerosis**							0.98 [0.97–0.99]

We also identified variables associated with the absence of AD coding: cancer, carcinoma in situ and benign tumor, diverticulosis, inflammation, rheumatoid arthritis, psoriasis, obesity, osteoarthritis, ischemic and non-ischemic heart disease.

## Discussion

Analysis of 137 variables concerning 1.4 million patients aged over 49 years, included in the PMSI with a 6-year perspective, revealed statistically significant associations between the onset of AD and about 20 explanatory variables. Some are well described (stroke, diabetes, female, alcohol, depression …) in literature [19], while others are still little explored (intracranial hypertension, epilepsy …)

Our study shows associations with a temporality criterion. These associations must be interpreted with caution. On the one hand, the dependent variable is the coding of a hospital diagnosis of AD: thus, some pathologies may be associated with a higher or lower diagnosis given the modalities of the stay (neurology or geriatrics stay, colonoscopic follow-up, ambulatory surgery, etc.). On the other hand, the explanatory variable may be interpreted as risk factors (increase in neural lesions), precipitating factors (earlier diagnosis) or confounding factors (common ground, early symptoms). For example, falling after age 80 can be a risk factor (head injury), a warning sign of AD or a confounding factor (diabetic neuropathy, Parkinson's disease, stroke …); falling is also a cause of hospitalization in geriatrics, where the assessment will likely include a cognitive assessment.

In our study, 55,997 patients had AD in 2014 (4.04%). Rate of AD in our study increases with age, as found in other studies: in France, the rates are about 6% of patients over 65, 18% of patients over 75 and up to 40% beyond 85 years (versus respectively 7.1%, 11.5% and 14.9% in our study) [20,21]. The main factors associated with AD in our study change according to the patient age. Some are well described, while others are still little explored.

In the literature, we find a similar association regarding diabetes [22–25], alcohol abuse [26], BMI < 18 kg/m^2^ [24,27], heart failure [28], depression [22,29,30], bipolar disorder [31], mental retardation or low level of education [22,26]. The prevalence of psychotic disorders or anxiety disorders in AD has been estimated about 34–40% [32,33]. Intoxications may be a confounding factor with psychiatric disorders or reflect attempted suicide [34,35]. Link between epilepsy and AD is described but poorly understood; the prevalence rate of dementia is estimated to be between 8.1 and 17.5% for epileptic patients and the prevalence rate of epilepsy is estimated to be between 1 and 9% for dement patients [36].

For the first time, we show a link between intracranial hypertension and Alzheimer's disease in the 60–65 age group and then in the 70 to 80 age group. This may seem surprising because intracranial hypertension rarely occurs in elderly patients due to age-related cerebral atrophy (including chronic subdural hematoma). Nevertheless, the hypothesis of a link between intracranial hypertension and Alzheimer's disease has already been formulated. Indeed, normal pressure hydrocephalus and head injuries (e. g. in boxers) can be accompanied by anatomopathological lesions similar to those of Alzheimer's disease [37,38]; repeated episodes of intracranial hypertension (during head injuries or conditions such as heart failure, sleep apnea syndrome or chronic obstructive pulmonary disease) may be a contributing, precipitating or triggering factor in Alzheimer's disease [39,40].

Several variables were not associated with the onset of AD in our study, unlike in some studies in the literature, as age-related hearing loss [41], hypertension [22,25,42], hypercholesterolemia [25,43], *Helicobacter pylori* infection [44], *Chlamydia trachomatis* infection [45], head injury [46], obesity [22,24,27] or essential tremor [47].

The main strength of our work is the sample size with over 55,000 AD patients in 2014 for whom we have reliable data recorded prospectively 6 years ago.

Data mining techniques and the large sample size make it possible to study a large number of variables and raise new hypotheses of risk factors. They also enable to confirm associations already described in certain sub-populations (according to age).

Our study has several limitations.

The main limitation of the reuse of the PMSI is the impossibility of returning to the source data and quality of coding. The use of the PMSI for activity-based pricing can also lead to overcoding of certain pathologies and undercoding codes without interest for pricing. Coding is the responsibility of the clinician and can sometimes be approximate: for example, it is possible to have a family history of cancer without it being coded in the database (weak interest for pricing); and in the case of intracranial hypertension, we cannot verify the data of papilledema or pressure of the lumbar puncture. Nevertheless, it is unlikely that there is a differential bias in favour of better or worse coding of intracranial hypertension or family history of cancer in patients rehospitalized with AD coding 6 years later.

Concerning quality of dependent variable, there is a strong correlation between a clinical ante-mortem diagnosis and a post-mortem diagnosis [48,49]. In our study, ICD-10 diagnosis of AD may have questionable accuracy and variation, or even be confounded by delirium in some cases considering the fact that most data points were from short-term hospital stays. Nevertheless, we have shown in preliminary studies that the diagnosis of AD is more reliable in PMSI in 2014 than in previous years, probably in connection with the proposal for new NINCDS-ADRA criteria [50–52].

Our study is a correlation analysis and not a retrospective cohort: we did not include competing risks (attrition, loss of sight, death, etc.) This selection strategy tends to create spurious negative associations between diagnoses of chronic conditions in 2008 and AD in 2014, since patients with neither chronic condition in 2008 nor AD in 2014 are more likely to be missing from the analysis set.

We used PMSI database excluding psychiatric hospitals and rehabilitation care centers: it could also have led to selection bias and this may explain the low rate of psychiatric disorders in our population.

We used PMSI database in 2008 and 2014. We have opted for this simple time management for several reasons: *primo*, this was allowed by the numbers of patients in our study; *secundo*, diagnosis of AD is more reliable in 2014 than in previous years [50–52] so incorporating the diagnosis of AD in 2012 or 2013 would probably have decreased the quality of this dependent variable; *tertio*, our objective was to identify risk factors rather than early symptoms, so we opted for the extreme years (2008 and 2014) allowed by the accreditation giving access to PMSI data at the time of analysis.

We have chosen a minimum age of 49 years in 2008 (55 years in 2014) because AD is rare before 55 years of age, and mainly concerns family cases. However, since genetics is not a study factor in the base of the short stay PMSI, we preferred to avoid the inclusion of these cases. Moreover, our maximum decline is 6 years, which does not really allow us to determine the first symptoms before the prodromal phase of 15 to 20 years.

Due to the extremely high impact of age on the onset of AD, we divided our sample into 5 years classes. This allowed us to identify the main explanatory variables according to different times in life and clinical situations: psychotic disorders after 60 years, intracranial hypertension, epilepsy or denutrition after 70 years… Nevertheless, the use of 5-year age intervals could produce spurious associations between diseases/conditions (such as AD) with strongly age-dependent prevalences (e.g. a spurious positive association with a condition such as undernutrition which is increasingly prevalent with older age). AD multifactorial nature adds the complexity of having too many confounding variables that are impossible to be adjusted for in the PMSI. It may be more informative to perform an age-stratified analysis on all data, testing for an effect of each condition on probability of AD and for an interaction between the condition and age.

It is not possible in the PMSI to adjust on certain variables known to be associated with AD: active smoking, sedentary lifestyle [22], diet [53], biological criteria as hyperhomocysteinemia [54], genetics (ApoE4e4 [55]) or medications as benzodiazepines [56–58].

We have identified pathologies inversely associated with AD diagnosis 6 years later: cancer, family history of cancer, inflammation, rheumatoid polyarthritis, psoriasis, etc. Some of these associations are cited in the literature as the inverse relationship with cancer [59,60] or rheumatoid arthritis [61–63]. As exposed above, they seem to be reasons for follow-up, i.e. iterative hospitalizations motivated mainly by the initial pathology, not necessarily leading to a coding of AD. A neuroprotective phenomenon of inflammation could also be evoked [64].

In conclusion, an analysis of 137 variables in the PMSI identified some well-known risk factors for AD, and highlighted a possible association with intracranial hypertension. Better knowledge of associations could lead to better targeting (identifying) at-risk patients, and better prevention of AD, in order to reduce its impact.
